# Supplementary material for: Immunofluorescence characterization of spinal cord dorsal horn microglia and astrocytes in horses
Source: PeerJ. 2017 Oct 27;5:e3965. doi: 10.7717/peerj.3965 (PMC5661433; doi:10.7717/peerj.3965)
Supplement: File S1 — Mean and standard deviation of astrocyte diameter, process length and thickness and distribution, and microglia cell area and distribution. [file peerj-05-3965-s001.docx]

**SUPPLEMENTARY FILES**


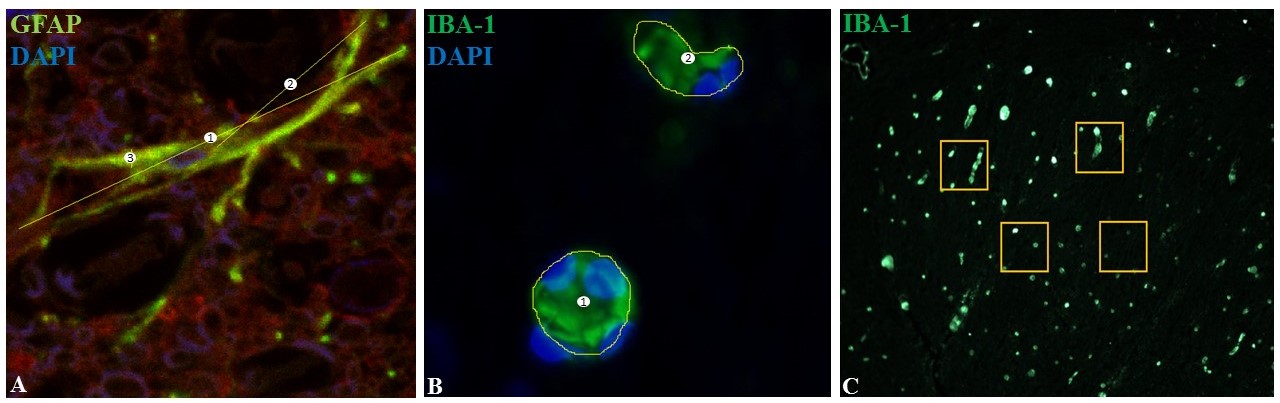


Morphological y organizational evaluation of microglia and astrocytes cells was performed within the dorsal horn using ImageJ software. (1) Cell diameter, (2) process length and (3) thickness of GFAP positive cells was evaluated. The diameter was determined measuring the longest axis in non-overlapping cells, with a cross sectional line through the nucleus (DAPI marked) and two endpoint branches. A line between the nucleus and the end of a major branch was traced to measure the process length, and the thickness of these cells was defined with a trace line between two parallel sides of the same branch (A). Cross sectional area of Iba-1 positive cells was defined using a surrounding line (1 and 2) in twenty cell bodies (DAPI marked), and then area was calculated (B). Cell counting was performed in each dorsal horn segment, using 30 square perimeter of 1,000 µm^2^ per segment for counting (C).
