# Supplementary material for: Immunofluorescence characterization of spinal cord dorsal horn microglia and astrocytes in horses
Source: PeerJ. 2017 Oct 27;5:e3965. doi: 10.7717/peerj.3965 (PMC5661433; doi:10.7717/peerj.3965)
Supplement: File S2 [file peerj-05-3965-s002.docx]

SUPPLEMENTARY FILE


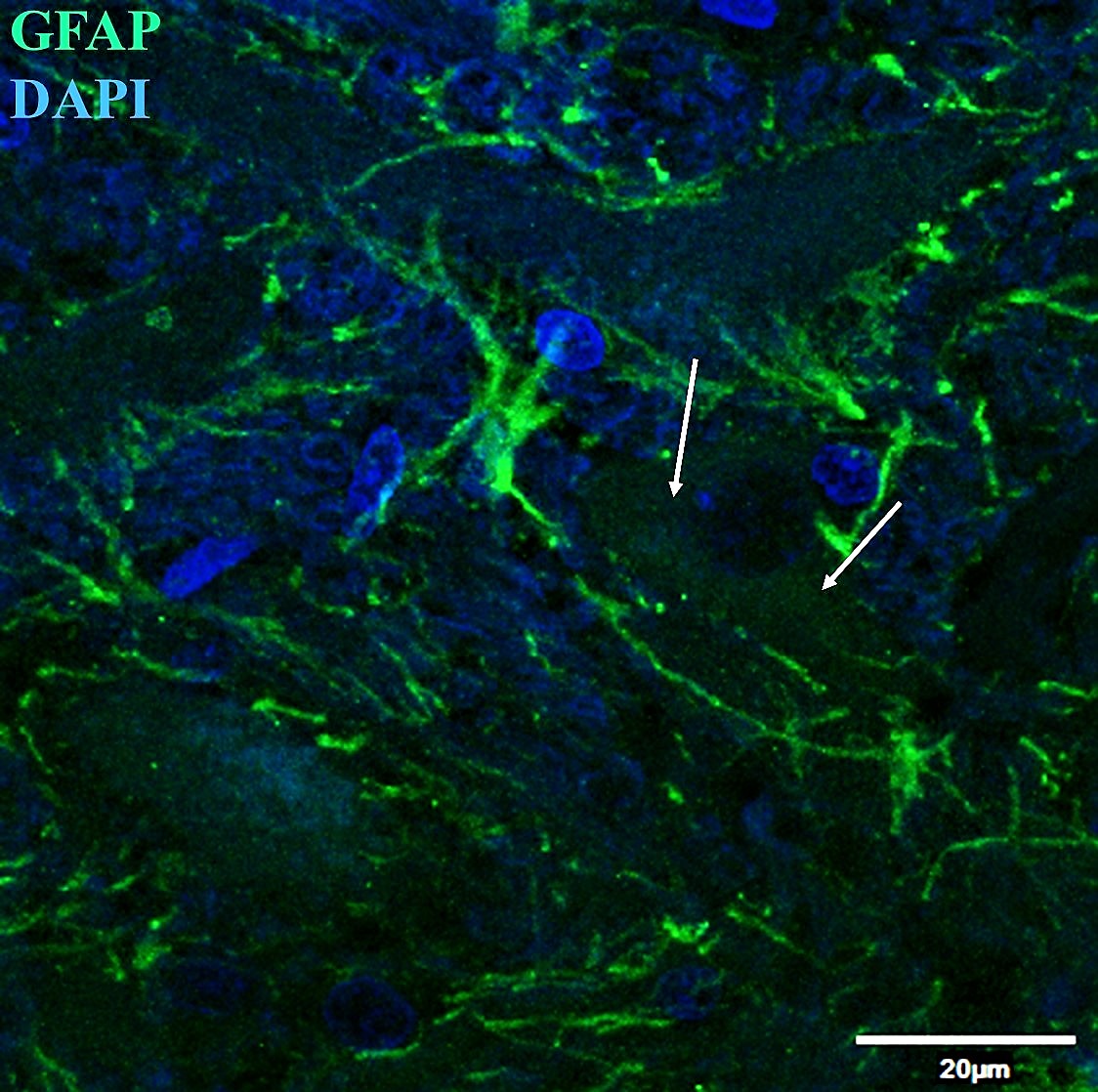


Astrocytes are often found in association with neighboring neurons (white arrows) in dorsal horn spinal cord in horses.
